# Supplementary material for: Bootstrap-quantile ridge estimator for linear regression with applications
Source: PLoS One. 2024 Apr 29;19(4):e0302221. doi: 10.1371/journal.pone.0302221 (PMC11057767; doi:10.1371/journal.pone.0302221)
Supplement: Attachment — Submitted filename: Response to PLOS ONEl.docx [file pone.0302221.s002.docx]

**Response to Editors Comments**

| **Editor Comments** |  | **Response on Comments** |
| --- | --- | --- |
| 1. Bootstrap generally considers two-third observations and hence produces underestimated results. Have you mentioned this as one of the demerits of your proposal.  2. Ridge-estimator is not used if there is correlated covariates in the model. Instead elastic-net is generally preferred for overcoming this issue. What is the rational of using Ridge estimator?  3. May need to cite some related papers: M.H.R. Khan, A. Bhadra and T. Howlader (2019). Stability Selection for Lasso, Ridge and Elastic Net Implemented with AFT Models. Statistical Applications in Genetics and Molecular Biology, 18(5), 20170001 M.H.R. Khan and J.E.H. Shaw (2016). Variable Selection for Survival Data with a Class of Adaptive Elastic Net Techniques. Statistics and Computing, 26(3): 725-741 M.H.R. Khan (2018). On The Performance of Adaptive Pre-processing Technique in Analysing High-dimensional Censored Data. Biometrical Journal, 60(4): 687-702.,. |  | As suggested by Efron, B., & Tibshirani, R. J. (1994). An introduction to the bootstrap. CRC press, we have considered the bootstrap samples with replacement and as of original sample size.  Unlike, elastic-net and other lasso type methods, ridge regression retains all the candidate variables. So, it is mainly used in situations where we face ill-conditioned problem and least squares estimation is imprecise or impossible. For some optimal choice of ridge parameter, the ridge regression minimizes the estimation variance at the cost of bias.  As per your suggestion, relevant citations are included. |

**Response to Reviewers**

**Reviewer 1:**

| **Reviewer Comments** |  | **Response on Comments** |
| --- | --- | --- |
| 1. I think that some recent papers related to "the ridge estimator" should be mentioned such as: - DOI: 10.3390/stats3040033; DOI:10.3389/fams.2022.952142; DOI:10.1080/03610918.2021.1960373; DOI: 10.1016/j.sciaf.2022.e01372; DOI:10.1080/03610918.2021.1939374.  2. In the simulation study, the author should use more different sample sizes, such as n>100. Moreover, it is very important to display the 'bias' amount of different biased estimators in the simulation study.  3. In application (table 8), what are the values of k's used for?  4. The author should put "datasets" that are used in the section of applications in a "Supporting Information File".  5. In section of conclusion, add a sentence about future work.  6. There are some grammatical errors in the paper. The author needs to carefully review the full text. |  | As per your suggestion, relevant citations are included.  Case of n=200 is included in the study. For this purpose, Table 6 is added in the manuscript.  Now the value of k’s are added in the table.  We have now used two real-life published datasets tobacco data and hospital manpower data set (Myers, 1990). These datasets are also available as internal datasets in R. R Core Team (2022). R: A language and environment for statistical computing. R Foundation for Statistical Computing, Vienna, Austria. URL https://www.R-project.org/.  A sentence is added showing future work.  The draft is extensively reviewed and revised in the light of reviewer’s comments. |
|  |  |  |

**Reviewer 2:**

| **Reviewer Comments** |  | **Response on Comments** |
| --- | --- | --- |
|  |  |  |
| 1. The novelty of the article must be highlighted in relation to the existing literature. Many articles related to this topic has been published in the literature so what is new? 2. Why MSE is used to assess the performance? What is the theoretical justification of the proposed method. It is suggested to the authors to derive the properties of the proposed estimators, especially large sample properties. 3. What is the theoretical justification of the proposed method. It is suggested to the authors to derive the properties of the proposed estimators, especially large sample properties. 4. How the initial values are selected? 5. A new real data set should be used. 6. How the initial values are taken to initialized the algorithm in real data case? 7. Literature review must be updated by including all recent contribution in ridge regression. 8. The complete computational code should be submitted to verify the reproducibility. |  | Our core motivation is to show that use of bootstrapping can improve the efficiency of any existing ridge estimator. Unlike Lasso-type methods, ridge regression is not capable of variable selection. So, the focus is to reduce the estimation error.  The proposed methodology generally improves the efficiency of estimators for all considered choices of sample size, error variance, dimension and multicollinearity level, so it can be considered robust option. So the main novelty of our work is the improvement of any ridge estimator by using an appropriate quantile of its bootstrap distribution.  The objective is to reduce error in the estimation of regression coefficients. MSE is widely used criterion in this regard (Cochran 2007). Gauss in 1809 suggested MSE as the most relevant criterion for the choice among estimators. There is a long list of related research work in which the same criterion has been considered see e,g.  [Hoerl and Kennard (1970](https://www.sciencedirect.com/science/article/pii/S0264999311000484?casa_token=HKPjc0DfD7UAAAAA:9q5PbNFz-h0431HYY4NoZnViF-p8jzDeAtYslKfWHTT_UFy9q6FGZvgYEwW3zhAaNXi6dhRjDw#bb0030)), [Hoerl et al. (1975](https://www.sciencedirect.com/science/article/pii/S0264999311000484?casa_token=HKPjc0DfD7UAAAAA:9q5PbNFz-h0431HYY4NoZnViF-p8jzDeAtYslKfWHTT_UFy9q6FGZvgYEwW3zhAaNXi6dhRjDw#bb0035)), [McDonald and Galarneau (1975](https://www.sciencedirect.com/science/article/pii/S0264999311000484?casa_token=HKPjc0DfD7UAAAAA:9q5PbNFz-h0431HYY4NoZnViF-p8jzDeAtYslKfWHTT_UFy9q6FGZvgYEwW3zhAaNXi6dhRjDw#bb0060)), [Lawless and Wang (1976](https://www.sciencedirect.com/science/article/pii/S0264999311000484?casa_token=HKPjc0DfD7UAAAAA:9q5PbNFz-h0431HYY4NoZnViF-p8jzDeAtYslKfWHTT_UFy9q6FGZvgYEwW3zhAaNXi6dhRjDw#bb0050)), [Dempster et al. (1977](https://www.sciencedirect.com/science/article/pii/S0264999311000484?casa_token=HKPjc0DfD7UAAAAA:9q5PbNFz-h0431HYY4NoZnViF-p8jzDeAtYslKfWHTT_UFy9q6FGZvgYEwW3zhAaNXi6dhRjDw#bb0015)), [Gibbons (1981](https://www.sciencedirect.com/science/article/pii/S0264999311000484?casa_token=HKPjc0DfD7UAAAAA:9q5PbNFz-h0431HYY4NoZnViF-p8jzDeAtYslKfWHTT_UFy9q6FGZvgYEwW3zhAaNXi6dhRjDw#bb0020)), [Kibria, (2003](https://www.sciencedirect.com/science/article/pii/S0264999311000484?casa_token=HKPjc0DfD7UAAAAA:9q5PbNFz-h0431HYY4NoZnViF-p8jzDeAtYslKfWHTT_UFy9q6FGZvgYEwW3zhAaNXi6dhRjDw#bb0045)), [Khalaf and Shukur (2006](https://www.sciencedirect.com/science/article/pii/S0264999311000484?casa_token=HKPjc0DfD7UAAAAA:9q5PbNFz-h0431HYY4NoZnViF-p8jzDeAtYslKfWHTT_UFy9q6FGZvgYEwW3zhAaNXi6dhRjDw#bb0040)), [Alkhamisi et al. (2006](https://www.sciencedirect.com/science/article/pii/S0264999311000484?casa_token=HKPjc0DfD7UAAAAA:9q5PbNFz-h0431HYY4NoZnViF-p8jzDeAtYslKfWHTT_UFy9q6FGZvgYEwW3zhAaNXi6dhRjDw#bb0010)), [Alkhamisi and Shukur (2008](https://www.sciencedirect.com/science/article/pii/S0264999311000484?casa_token=HKPjc0DfD7UAAAAA:9q5PbNFz-h0431HYY4NoZnViF-p8jzDeAtYslKfWHTT_UFy9q6FGZvgYEwW3zhAaNXi6dhRjDw#bb0005)), Alheety and Kibria(2009),[Muniz and Kibria (2009](https://www.sciencedirect.com/science/article/pii/S0264999311000484?casa_token=HKPjc0DfD7UAAAAA:9q5PbNFz-h0431HYY4NoZnViF-p8jzDeAtYslKfWHTT_UFy9q6FGZvgYEwW3zhAaNXi6dhRjDw#bb0065)),), Dorugade and Kashid (2010), Månsson et al.(2010), Muniz et al. (2012),Ertaş et al. (2013), Asar et al. (2014), Ayinde et al. (2015),Kibria and Banik (2016), Asar and Genc (2017). Kaçıranlar, and Güler (2017), Ertaş (2018), Lukman and Olatunji(2018),Qasim et al. (2019), Göktaş et al.(2020), Kibria and Lukman (2020), Suhail et al. (2020), Babar et al.(2021), Perveen and Suhail(2021),Yasin et al. (2021), Lukman et al. (2020), Babar and Chand(2022), Shabbir et al.(2022) , Dar et al. (2023) among others.  A section on theoretical justification is added in the manuscript.  We have used the grid search the starting quantile value is 0.1 with step size of 0.1.  Many thanks for your suggestion. Noting it, we have now considered published datasets which are widely studied in ridge regression literature. It will help to have a better comparison with the existing literature and reference for future researches.    In simulation study, the data were generated under various scenario as described in the manuscript. For the extensive evaluation of proposed estimator, the qunatile level was initiated at 0.1 and optimal point was searched using the performance metric.  Recent literature is added.  As per your instruction, the R code is attached. |
|  |  |  |
