# Supplementary material for: Bootstrap-quantile ridge estimator for linear regression with applications
Source: PLoS One. 2024 Apr 29;19(4):e0302221. doi: 10.1371/journal.pone.0302221 (PMC11057767; doi:10.1371/journal.pone.0302221)
Supplement: Attachment — Submitted filename: Response to PLOS ONE 2.docx [file pone.0302221.s003.docx]

**Response to Reviewers**

**Reviewer 2:**

| **Reviewer Comments** |  | **Response to the Comments** |
| --- | --- | --- |
| 1. There is no attachment showing the complete computational code to verify the reproducibility.  2. The source of 'Hospital manpower data' is not mentioned.  3. Why is not MAPE or MAE used rather than MSE?  5. The theoretical justification of bootstrap is available in books. Please cite an appropriate reference.  6. Can we consider a situation of n=10, and p=15? |  | As per your suggestion, the complete code is attached.  The data is taken from Myers (1990) and is mentioned in the manuscript. Also, it is available in R library (genridge).  The objective is to reduce error in the estimation of regression coefficients. MSE is a widely used criterion in this regard (Cochran 2007). Gauss in 1809 suggested MSE as the most relevant criterion for the choice among estimators. There is a long list of related research work in which the same criterion has been considered see e,g.  [Hoerl and Kennard (1970](https://www.sciencedirect.com/science/article/pii/S0264999311000484?casa_token=HKPjc0DfD7UAAAAA:9q5PbNFz-h0431HYY4NoZnViF-p8jzDeAtYslKfWHTT_UFy9q6FGZvgYEwW3zhAaNXi6dhRjDw#bb0030)), [Hoerl et al. (1975](https://www.sciencedirect.com/science/article/pii/S0264999311000484?casa_token=HKPjc0DfD7UAAAAA:9q5PbNFz-h0431HYY4NoZnViF-p8jzDeAtYslKfWHTT_UFy9q6FGZvgYEwW3zhAaNXi6dhRjDw#bb0035)),  [Lawless and Wang (1976](https://www.sciencedirect.com/science/article/pii/S0264999311000484?casa_token=HKPjc0DfD7UAAAAA:9q5PbNFz-h0431HYY4NoZnViF-p8jzDeAtYslKfWHTT_UFy9q6FGZvgYEwW3zhAaNXi6dhRjDw#bb0050)),  [Gibbons (1981](https://www.sciencedirect.com/science/article/pii/S0264999311000484?casa_token=HKPjc0DfD7UAAAAA:9q5PbNFz-h0431HYY4NoZnViF-p8jzDeAtYslKfWHTT_UFy9q6FGZvgYEwW3zhAaNXi6dhRjDw#bb0020)), [Kibria, (2003](https://www.sciencedirect.com/science/article/pii/S0264999311000484?casa_token=HKPjc0DfD7UAAAAA:9q5PbNFz-h0431HYY4NoZnViF-p8jzDeAtYslKfWHTT_UFy9q6FGZvgYEwW3zhAaNXi6dhRjDw#bb0045)), [Khalaf and Shukur (2006](https://www.sciencedirect.com/science/article/pii/S0264999311000484?casa_token=HKPjc0DfD7UAAAAA:9q5PbNFz-h0431HYY4NoZnViF-p8jzDeAtYslKfWHTT_UFy9q6FGZvgYEwW3zhAaNXi6dhRjDw#bb0040)),  Alheety and Kibria(2009),[Muniz and Kibria (2009](https://www.sciencedirect.com/science/article/pii/S0264999311000484?casa_token=HKPjc0DfD7UAAAAA:9q5PbNFz-h0431HYY4NoZnViF-p8jzDeAtYslKfWHTT_UFy9q6FGZvgYEwW3zhAaNXi6dhRjDw#bb0065)), Muniz et al. (2012), Asar et al. (2014), Kibria and Banik (2016), Asar and Genc (2017). Kaçıranlar, and Güler (2017), Lukman and Olatunji (2018), Qasim et al. (2019), Göktaş et al. (2020), Kibria and Lukman (2020), Suhail et al. (2020), Babar et al. (2021), Perveen and Suhail (2021), Lukman et al. (2020), Babar and Chand (2022), Shabbir et al. (2022), Dar et al. (2023) among others.  However, MAE and MAPE are used in prediction estimation which is not the scope of our study.  As per suggestion now proper citation is included in the paper.  This is the scenario of high dimensional settings which is not the focus of this paper. |
|  |  |  |
